# *ISPD* mutations account for a small proportion of Italian Limb Girdle Muscular Dystrophy cases

**DOI:** 10.1186/s12883-015-0428-8

**Published:** 2015-09-24

**Authors:** Francesca Magri, Irene Colombo, Roberto Del Bo, Stefano Previtali, Roberta Brusa, Patrizia Ciscato, Marina Scarlato, Dario Ronchi, Maria Grazia D’Angelo, Stefania Corti, Maurizio Moggio, Nereo Bresolin, Giacomo Pietro Comi

**Affiliations:** Dino Ferrari Centre, Department of Neurological Sciences, University of Milan, I.R.C.C.S. Foundation Cà Granda, Ospedale Maggiore Policlinico, via F. Sforza 35, 20122 Milan, Italy; Neuromuscular and Rare Disease Unit, Department of Neuroscience, Foundation IRCCS Ca’ Granda Ospedale Maggiore Policlinico, University of Milan, via F. Sforza 35, 20132 Milan, Italy; Inspe, Division of Neuroscience, San Raffaele, Via Olgettina 60, Milan, Italy; IRCCS E. Medea, Bosisio Parini, Italy

**Keywords:** Isoprenoid synthase domain-containing gene, GDP-mannose pyrophosphorylase B, Limb Girdle Muscular Dystrophy, α-dystroglycan glycosylation

## Abstract

**Background:**

Limb Girdle Muscular Dystrophy (LGMD), caused by defective α-dystroglycan (α-DG) glycosylation, was recently associated with mutations in Isoprenoid synthase domain-containing (*ISPD*) and GDP-mannose pyrophosphorylase B (*GMPPB*) genes. The frequency of *ISPD* and *GMPPB* gene mutations in the LGMD population is unknown.

**Methods:**

We investigated the contributions of *ISPD* and *GMPPB* genes in a cohort of 174 Italian patients with LGMD, including 140 independent probands. Forty-one patients (39 probands) from this cohort had not been genetically diagnosed. The contributions of *ISPD* and *GMPPB* were estimated by sequential α-DG immunohistochemistry (IHC) and mutation screening in patients with documented α-DG defect, or by direct DNA sequencing of both genes when muscle tissue was unavailable.

**Results:**

We performed α-DG IHC in 27/39 undiagnosed probands: 24 subjects had normal α-DG expression, two had a partial deficiency, and one exhibited a complete absence of signal. Direct sequencing of *ISPD* and *GMPPB* revealed two heterozygous *ISPD* mutations in the individual who lacked α-DG IHC signal: c.836-5 T > G (which led to the deletion of exon 6 and the production of an out-of-frame transcript) and c.676 T > C (p.Tyr226His). This patient presented with sural hypertrophy and tip-toed walking at 5 years, developed moderate proximal weakness, and was fully ambulant at 42 years. The remaining 12/39 probands did not exhibit pathogenic sequence variation in either gene.

**Conclusion:**

*ISPD* mutations are a rare cause of LGMD in the Italian population, accounting for less than 1 % of the entire cohort studied (*FKRP* mutations represent 10 %), while *GMPPB* mutations are notably absent in this patient sample. These data suggest that the genetic heterogeneity of LGMD with and without α-DG defects is greater than previously realized.

**Electronic supplementary material:**

The online version of this article (doi:10.1186/s12883-015-0428-8) contains supplementary material, which is available to authorized users.

## Background

Limb girdle muscular dystrophies (LGMDs) are a heterogeneous group of inherited progressive muscle disorders characterized by progressive shoulder and pelvic girdle muscle weakness variably associated with cardiac, respiratory, and cognitive involvement [1]. The number of genes involved in these disorders has exponentially increased in recent years, and now up to 30 different forms have been described, inherited both with autosomal dominant (7 forms) and autosomal recessive (23 forms) patterns [2].

In particular, a high number of genes involved in α-dystroglycan (α-DG) glycosylation have been associated with LGMD. α-DG is a highly glycosylated core component of the dystrophin glycoprotein complex, and forms a link between the sarcolemma and the extracellular matrix [3]. To date, mutations in 14 genes [4–18], all coding for putative or demonstrated glycosyltransferase, have been associated with muscular dystrophies (referred to as secondary dystroglycanopathies) [19], while only a couple of cases have been associated with mutations in *DAG1*, the gene that encodes both α-dystroglycan and β-dystroglycan [20, 21]. Dystroglycanopathies are characterised by a broad variety of clinical phenotypes, ranging from congenital muscular dystrophy (CMD), with or without brain and eye involvement as Walker-Warburg syndrome (WWS) and muscle eye brain disease (MEB), to LGMD, as summarised by Godfrey et al. [22]. Mutations in most of these genes are mainly associated with severe or congenital conditions, with few notable exceptions: *FKRP* mutations account for a variable proportion of LGMD depending on ethnic background (from 6 % in the Italian population [1, 23] to 38 % in the Danish population [24]). On the other hand, LGMD phenotypes caused by mutations in *POMT1* (LGMD2K) [25], *FKTN* (LGMD2L) [26], *POMT2* (LGMD2N) [27], *POMGNT1* (LGMD2O) [22], *DAG1* [21], and *DPM3* [10] have been reported in a very limited number of patients.

*ISPD*, a gene located on chromosome 7p21, encodes the Isoprenoid synthase domain-containing protein and has been implicated in the initial step of the O-mannosylation of α-DG. Mutations in this gene were first identified within the most severe spectrum of dystroglycanopathies, WWS and MEB cases [13, 14], although more recently they have also been associated with milder phenotypes [28, 29]. In a paediatric cohort of dystroglycanopathies with British and Turkish background, *ISPD* mutations have been found to cause LGMD in seven probands, including four LGMD cases with normal cognitive development (LGMD – no MR); two LGMD cases with cerebellar involvement (LGMD-CRB); and one case of LGMD with mental retardation, but without structural brain abnormalities (LGMD-MR) [28]. *ISPD* mutations were also detected in two Italian LGMD families that presented with disease onset during the first two decades of life, late motor impairment, and no functional or structural brain involvement [28]. Muscle biopsy revealed dystrophic features and α-DG reduction at immunohistochemistry [28, 29]. According to this description, forms of LGMD caused by mutations in *ISPD* are described as LGMD2S [2]. Intra-familial variability has also been described [30].

*ISPD* mutations account for 9–11 % of the most severe dystroglycanopathy variants (comprising CMD, WWS, and cobblestone lyssencephaly) in three large cohorts from different ethnic backgrounds [13, 14, 17]. The prevalence of *ISPD* mutations has not yet been estimated in LGMD cohorts: at this time, only 12 patients with *ISPD* mutation and this phenotype have been described. Similar considerations apply to disease phenotypes caused by *GMPPB* mutations, initially shown to be causally linked to MEB/FCMD-like syndrome [16, 31], and more recently to a wider phenotypic spectrum that includes infantile phenotypes with mental retardation [16] and adult-onset LGMD with normal cognition [32].

The aim of this study is to establish the prevalence of *ISPD* and *GMPPB* mutations within an Italian cohort of LGMD patients.

## Methods

### Patient selection and characterization

From a cohort of 174 Italian LGMD patients (140 families), all followed at a single neuromuscular centre, we selected 41 patients (39 families) without a molecular diagnosis. Written informed consent was obtained (and preserved in original) from all patients or their caregivers at first evaluation, with explicit consent to future use for research purposes, in accordance with the Declaration of Helsinki. This protocol was approved by the Research Ethics Board of IRCCS Foundation Ca’ Granda Ospedale Maggiore Policlinico. The patients have been previously screened for the following genes: *MYOT, LMNA, CAV3, DNAJB6,* and *TNPO3,* if autosomal dominant transmission was supported by family history*; CAPN3, DYSF, SGCA, SGCB, SGCG, SGCD, FKRP, ANO5, FKTN,* and *LAMA2* in sporadic or autosomal recessive cases.

LARGE, POMT1, POMT2, POMGnT1 were also screened in selected cases.

Patients were defined as affected with LGMD if they fulfilled the following criteria: clinical phenotype characterised by progressive muscle weakness and wasting affecting primarily the shoulder girdle and pelvic muscles, in keeping with the diagnostic criteria for LGMD [33]; and dystrophic features at muscle biopsy. All patients had undergone systematic clinical characterisation, including comprehensive neurological [Medical Research Council (MRC) and functional scales], cardiac (electrocardiogram and echocardiogram), and respiratory (spirometry and nocturnal saturimetry) assessments. Data about clinical and familial history were also collected. All specimens were obtained from the Skeletal Muscle, Peripheral Nerve, DNA and Cell Line Bank of the Neuromuscular Unit, Fondazione IRCCS Ca’ Granda Ospedale Maggiore Policlinico, University of Milan. Written informed consent was obtained and preserved in the original form from all individuals or their caregivers when primary diagnostic procedures were performed, with explicit consent for future use for research purposes, according to the Declaration of Helsinki.

### Muscle biopsy analysis

All probands had previously undergone a skeletal muscle biopsy during the period between 1975 and 2014. Muscle samples were frozen in isopentane, cooled in liquid nitrogen, and stained histochemically according to standard procedures [34]. We reviewed muscle biopsies from cases without a genetic characterization, which included data about α-DG immunohistochemical (IHC) analysis (performed with the clone VIA4-1; Merck Millipore, UK). In muscle samples in which α-DG IHC had not been conducted previously, this study was performed using the antibody cited above if a muscle tissue sample was available.

### Molecular analysis

Genomic DNA was extracted from peripheral blood samples according to standard procedures (Flexi Gene DNA Handbook, Qiagen).

*ISPD* and *GMPPB* analysis were performed in patients who exhibited α-DG deficiency at muscle IHC and in patients who had LGMD inclusion criteria, but whose muscle sample was unavailable at the time of this investigation. Mutations in FKRP, the most common LGMD gene involved in α-DG glycosylation [1, 32, 35], were also ruled out for the first group of patients.

All exons and flanking intronic regions were directly sequenced using an ABI PRISM 3100 XL Genetic Analyzer (Applied Biosystems) and previously published primer [13, 16].

The pathogenic nature of new mutations was confirmed by screening 100 healthy control subjects. The intronic mutation that leads to abnormal mRNA splicing was investigated through transcript analysis. The parental origin of each mutation was assessed through analysis of parental genomic DNA, when available. Amino acid conservation was confirmed by comparison with sequences from different species.

We isolated mRNA from muscle tissue using Eurozol; the cDNA was produced through reverse transcription polymerase chain reaction (Ready-To-Go RT-PCR kit, Amersham Pharmacia) and analyzed by amplification, cloning, and sequencing. Mutations were named according to the Leiden Muscular Dystrophy database (www.dmd.nl).

## Results

### Relative frequency of ISPD and GMPBB mutations in an Italian cohort

**Fig. 1:**
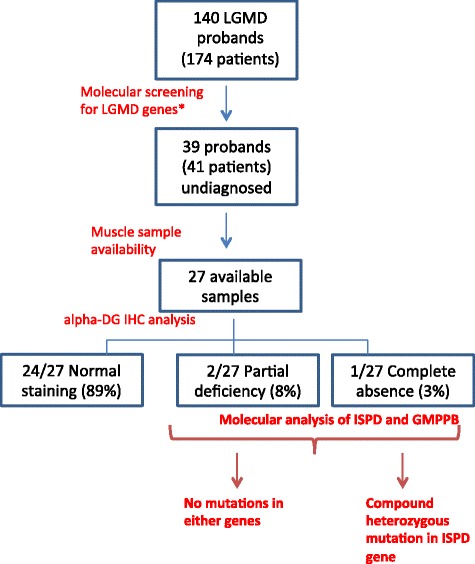
Patient selection for *ISPD* analysis and mutation rate. α-DG IHC analysis in undiagnosed patients revealed that α-DG was reduced in 3/27 individuals (11 % of our sample). One of three patients exhibited an *ISPD* mutation. *Molecular screening was performed according to clinical and bioptical characteristics

Among a single-centre cohort of 174 Italian LGMD patients (140 probands), 41 patients (39 probands) were without a genetic diagnosis. For 12/39 probands, we directly analyzed *ISPD* and *GMPPB* because muscle biopsy samples were unavailable for α-DG IHC analysis: none contained mutations. For 27/39 probands with available muscle biopsy, we performed α-DG IHC analysis, followed by *ISPD* and *GMPPB* sequencing in cases that exhibited any glycosylation defect: of 27 individuals, 24 had normal muscle α-DG expression, two had a partial deficiency, and one exhibited a complete absence of signal. The latter three cases were analysed for *FKRP* and GMPPB showing wild-type sequences. Only one of them had *ISPD*-causative mutations. Noteworthy, this latter proband was the only case with a muscle biopsy that exhibited complete absence α-DG labelling (Fig. 1).

**Fig. 2:**
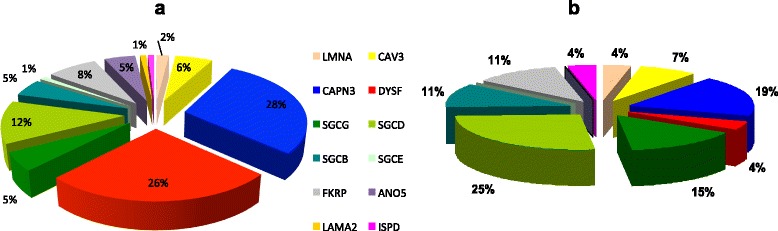
Relative frequency of *ISPD* mutations. **a** The relative frequency of different mutations in the entire sample. *ISPD* mutations account for approximately 1 % of the probands. **b** The relative frequencies of different mutations in the sample of patients younger than 10 years old. *ISPD* mutations account for 4 % of the probands

Including this new case, the 102/140 probands with a confirmed genetic diagnosis of LGMD had mutations in the following genes: 2/102 (2 %) *LMNA*, 6/102 (6 %) *CAV3*, 29/102 (28 %) *CAPN3*, 27/102 (25 %) *DYSF*, 5/102 (5 %) *SGCG*, 12/102 (12 %) *SGCD*, 5/102 (5 %) *SGCB*, 1/102 (1 %) *SGCE*, 8/102 (8 %) *FKRP* 5/102 (5 %) *ANO5*, and 1/102 (1 %) *LAMA2*. The molecular data of these patients are listed in a separate table [see Additional file 1]. Overall, *ISPD* mutations account for 0.9 % of genetically characterized LGMD probands (Fig. 2a). Among our paediatric LGMD cohort, defined as patients with disease onset before 10 years of age (33 patients; 27 probands) *ISPD* mutations account for 4 % of cases. Relative frequencies in the paediatric onset population are indicated in Fig. 2b; sarcoglycanopathies (51 %), calpainopathies (19 %), and LGMD2I (11 %) are the most frequent forms.

### Molecular analysis

**Fig. 3:**
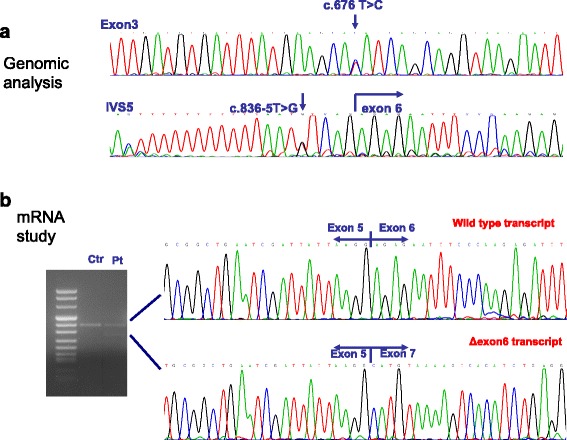
*ISPD* molecular analysis. **a** Electropherograms depicting the missense mutation c.676 T > C and the intronic substitution c.836-5 T > G. **b** mRNA analysis reveals the production of two different transcripts: the wild-type transcript, and a shorter transcript that corresponds to an isoform with a lower molecular weight. cDNA cloning demonstrated that the latter isoform corresponds to an isoform lacking exon 6

The *ISPD*-mutated patient carried two compound heterozygous mutations (Fig. 3a). The first mutation (c.676 T > C; p.Tyr226His) was a missense substitution in exon 3. Vuillaumier-Barrot et al. reported this mutation previously [17] in a heterozygotic foetal case affected with cobblestone lissencephaly; it was associated with a large deletion involving exons 4 through 6. The concerned amino acid is highly conserved among species. The second mutation (c.836-5 T > G) was a novel intronic substitution in IVS5. Its effect was investigated through transcript analysis, which revealed the production of two different transcripts: the wild-type transcript, and a shorter transcript corresponding to an isoform with lower molecular weight. cDNA cloning demonstrated that the smaller isoform corresponds to a transcript in which the deletion of exon 6 results in the production of an out-of-frame transcript (Fig. 3b). No alternative splicing could be demonstrated because the full-length transcript is completely encoded by the other allele, which contains a missense mutation.

### Clinical findings

**Fig. 4:**
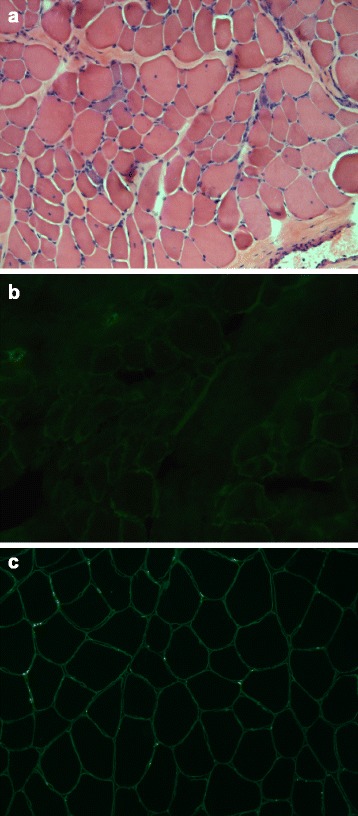
Muscle biopsy analysis. Quadriceps muscle biopsy of the patient with a mutation in the *ISPD* gene, performed at 4 years of age. Diffuse variation in fiber size, basophilic regenerating fibers, increased internal nuclei, hypercontracted fibers, and moderate increase of endomysial connective tissue were shown, characterising a typical dystrophic pattern (**a** haematoxylin and eosin, 20×). In the same patient, α-DG IHC (**b** clone VIA4-1, 20×) revealed absence of signal (**c** control muscle, 20×)

The only patient with a mutation in *ISPD* was a male who had been followed in our neuromuscular unit since he was 5 years old. He was referred to our centre in the late 1970s because of tip-toed walking. His family history was negative for neuromuscular conditions. Both parents were of Sicilian ancestry, and no consanguinity was reported. The patient’s prenatal history, delivery, and psycho-motor development were unremarkable. At the time of his first evaluation, neurological examination revealed only mild sural hypertrophy. Muscle strength was normal overall, with the exception of mild weakness at the pelvic girdle with positive Gowers’ sign. Creatine kinase levels ranged from 630 to 1200 IU/L (normal values <200 IU/L). Neurophysiological investigation revealed signs of mild myopathy at needle examination. The patient underwent a quadriceps muscle biopsy, which indicated a dystrophic process (Fig. 4). Considering these clinical and morphological findings, a diagnosis of Becker muscular dystrophy was initially supposed; however, later IHC analyses and Western blotting with monoclonal antibodies directed against dystrophin (Novocastra, 28 Newcastle Upon Tyne, UK) did not reveal any abnormalities. Furthermore, IHC analysis of caveolin-3 (Transduction Laboratories, Lexington, KY) and sarcoglycans (Transduction Laboratories, Lexington, KY) demonstrated normal staining. Dysferlin Western blot (Novocastra antibody) did not demonstrate any reduction of protein.

The patient’s motor performance has been stable since the age of 14 years, when the patient first complained of running difficulty and muscle cramps during exercise. Over the following decades, muscle weakness demonstrated a progressive course. At his last clinical examination, at the age of 42 years, he showed mild weakness at the neck flexors (MRC: 4) with moderate proximo-distal weakness at all four limbs (MRC: deltoids 3, brachial biceps 4, triceps 3, wrist extensors/flexors 4, sartorius 2, hip extra- and intra-rotators 3, quadriceps 3, tibialis anterior 4). He had universally decreased deep tendon reflexes, myopathic facies, bilateral scapular winging, and mild scoliosis. He walked with a waddling-type gait, and was able to climb stairs with double support. Ability to run had been lost (he walked 10 meters in 6.19 seconds). Regarding pulmonary involvement, spirometry indicated that his forced vital capacity was 3.35 L (71 %); nocturnal oxygen saturation was normal. His cardiac evaluation (echocardiogram) documented a right bundle branch block without any abnormalities of contractility. No cognitive impairment was observed (the patient acquired a degree in Engineering and worked as an engineer). We were unable to perform magnetic resonance imaging studies because of the patient’s severe claustrophobia.

## Discussion

Mutations in genes involved in α-DG glycosylation have been associated with a broad spectrum of disorders ranging from severe CMD to milder LGMD phenotypes [36]. An increasing number of genes involved in these disorders have been discovered recently, enlarging the spectrum of molecular heterogeneity of dystroglycanopathies. Among them, *ISPD* and *GMPPB* appear to follow *FKRP* as relevant genes in the LGMD population, according to earlier reports [16, 28].

It should be kept in mind that variations in relative frequency for a gene may be population-dependent. This is the case for the *FKRP* gene, which accounts for approximately 6 % of LGMD in Italian patients [1, 23], compared with 19 % among the British population [37] and 38 % among the Danish population [24]. Regarding other genes responsible for secondary dystroglycanopathies, only sporadic cases with LGMD phenotype have been reported thus far [10, 16, 21, 25–28]. In particular, *ISPD* mutations appear to be responsible for a relatively high proportion of dystroglycanopathies within the most severe clinical spectrum [13, 14, 17], although they have also been described in a few LGMD cases [28–30].

We analysed a large cohort of Italian LGMD patients in order to estimate the frequency of *ISPD* and *GMPPB* mutations and their associated clinical picture. In our LGMD sample, only a small proportion of undiagnosed cases (3/27, 11 %) exhibited reduction of α-DG staining. This finding suggests that if we exclude mutations in the *FKRP* gene, the other forms associated with defects of α-DG glycosylation are much more rare. Forms of LGMD caused by mutations in *ISPD* were also rare overall in our cohort, as they represent 0.9 % of genetically defined cases. This proportion increases to 4 % if we consider the group of patients with onset before 10 years of age (which is mainly patients with sarcoglycanopathies, calpainopathies, and LGMD2I). Interestingly, *GMPPB* mutations were absent from our cohort.

Overall, our patient carrying *ISPD* mutations presented a very mild LGMD phenotype compared with other cases described in the literature. He presented with early onset at 5 years of age with abnormal gait on tiptoe, and complained of his first motor limitation (impairment of his ability to run) at 14 years of age. Muscle weakness demonstrated a slowly progressive course with preserved independent ambulation at 42 years of age. Motor performances in the previously reported *ISPD-*mutated cases were variable, ranging from supported standing to independent running [29]; however, loss of independent ambulation (or ambulation for very short distances) has been reported universally in patients who had their last follow-up in adult age [28, 29]. In particular, the four cases of LGMD without central involvement described by Cirak et al. [29] presented a more severe Duchenne-like phenotype: they have early onset (1.5 to 3 years), higher creatine kinase levels, and severe progression (3 of 4 were non-ambulant at 12 years of age). Regarding cardiac impairment in patients with mutated *ISPD*, one adult patient exhibited a cardiac conduction defect in a likely history of previous myocardic ischemia [28], and three of four LGMD children described by Cirak et al. exhibited decreased contractility without any conduction defects [29]. Respiratory impairment has been described in a minority of patients affected with CMD with α-dystroglycan deficiency [36, 38]; however, decreased pulmonary volumes have been detected among both paediatric and adult patients with *ISPD* mutations [28, 29]. Interestingly, our patient did not exhibit any cardiac or respiratory involvement and he was fully ambulant in his forties, featuring the mildest symptoms on the *ISPD-*mutated spectrum reported thus far. Furthermore, our case confirms that absence of cognitive impairment is common in patients with *ISPD* mutations and LGMD phenotype [28, 29].

*ISPD* pathogenic mutations are generally located in the first exons of the gene. Furthermore, LGMD phenotypes are generally associated with milder mutations, such as missense and in-frame mutations, compared with CMD presentations.

In our patient, we detected two heterozygous mutations located in the first exons, namely one missense substitution and one intronic change that caused alteration of splicing and production of an out-of-frame transcript. The missense mutation had also been described in a foetal presentation with cobblestone lissencephaly, in association with a large deletion of three exons (exons 4 through 6) [17]. We can argue that our patient’s relatively mild phenotype is correlated with the compensatory action of other enzymes that have been implicated in α-DG glycosylation.

Immunohistochemical analysis revealed a complete absence of α-DG staining in our case. Among dystroglycanopathies, good correlation between α-DG staining and disease course was demonstrated in only a few forms (*POMT1*, *POMT2*, and *POMGnT1*-mutated cases), and is absent in patients with mutations in *FKRP* and *FKTN* [39]. As previously reported, α-DG labelling was severely reduced or absent in all patients with mutations in *ISPD*, irrespective of clinical severity [13, 14, 28, 29]. Our case, in whom clinical phenotype did not correlate with the absence of α-DG, further supports this lack of correlation.

## Conclusion

Overall, *ISPD* mutations are a rare cause of LGMD in the Italian population, and account for approximately 1 % of our entire cohort of genetically characterised LGMD (in comparison, *FKRP* mutations are responsible for up to 8 %). If we consider patients with paediatric onset, the frequency of LGMD2S increases to 4 % of molecularly diagnosed forms. At the time of this writing, no cases with adult onset have been reported. *GMPPB* mutations were absent in our cohort. Furthermore, reduction of α-DG staining is not frequent among LGMD cases; it accounts for only 11 % of biopsies from genetically undiagnosed patients. However, considering the increasing number of genes involved in α-DG glycosylation and the genetic overlap between congenital muscular dystrophies and LGMD, α-DG IHC analysis should be always performed in cases of undiagnosed LGMD in order to detect reduction of the protein level, which can then be investigated further.

## Additional file

Additional file 1:
**Molecular characterization of the LGMD cohort.** The table illustrates the detailed molecular data of the 102 molecularly diagnosed patients. (PDF 68 kb)

## Abbreviations

LGMD: Limb Girdle Muscular Dystrophy
α-DG: α-dystroglycan
CMD: Congenital muscular dystrophy
WWS: Walker-Warburg syndrome
MEB: Muscle eye brain disease
*FKRP*: *Fukutin related protein*
*ISPD*: Isoprenoid synthase domain-containing gene
GMPPB: GDP-mannose pyrophosphorylase CK, Creatine kinase
MRI: Magnetic Resonance Imaging
MRC scale: Medical Research Council Scale
IHC: Immunohistochemical
WB: Western blot
