# Supplementary material for: ISPD mutations account for a small proportion of Italian Limb Girdle Muscular Dystrophy cases
Source: BMC Neurol. 2015 Sep 24;15:172. doi: 10.1186/s12883-015-0428-8 (PMC4582941; doi:10.1186/s12883-015-0428-8)
Supplement: Additional file 1: — Molecular characterization of the LGMD cohort. The table illustrates the detailed molecular data of the 102 molecularly diagnosed patients. (PDF 68 kb) [file 12883_2015_428_MOESM1_ESM.pdf]

**Additional file 1 Molecular characterization of the LGMD cohort**										
**Pt N.**	**Sex**	**Gene**	**Allele 1**	**Nucleotide 1**	**Exon**	**Reference**	**Allele 2**	**Nucleotide 2**	**Exon**	**Reference**
1	F	LMNA	c.1567G>A	p.Gly523Arg	9	**New**	-	-	-	-
2.1	M	LMNA	c.1489-2A>C	Spl?	IVS8	**New**	-	-	-	-
2.2	M	LMNA	c.1489-2A>C	Spl?	IVS8	**New**	-	-	-	-
3.1	M	CAV3	c.290_292del	p.Phe97del	2	Cagliani R., 2003	-	-	-	-
3.2	F	CAV3	c.290_292del	p.Phe97del	2	Cagliani R., 2003	-	-	-	-
3.3	M	CAV3	c.290_292del	p.Phe97del	2	Cagliani R., 2003	-	-	-	-
3.4	F	CAV3	c.290_292del	p.Phe97del	2	Cagliani R., 2003	-	-	-	-
3.5	M	CAV3	c.290_292del	p.Phe97del	2	Cagliani R., 2003	-	-	-	-
3.6	M	CAV3	c.290_292del	p.Phe97del	2	Cagliani R., 2003	-	-	-	-
3.7	M	CAV3	c.290_292del	p.Phe97del	2	Cagliani R., 2003	-	-	-	-
3.8	F	CAV3	c.290_292del	p.Phe97del	2	Cagliani R., 2003	-	-	-	-
3.9	M	CAV3	c.290_292del	p.Phe97del	2	Cagliani R., 2003	-	-	-	-
3.10	M	CAV3	c.290_292del	p.Phe97del	2	Cagliani R., 2003	-	-	-	-
4.1	M	CAV3	c.136G>A	p.Ala46Thr	2	Hermann R., 2000	-	-	-	-
4.2	F	CAV3	c.136G>A	p.Ala46Thr	2	Hermann R., 2000	-	-	-	-
5.1	F	CAV3	c.80G>A	p.Arg27Gln	1	Carbone I, 2000	-	-	-	-
6.1	M	CAV3	c.169G>A	p.Val57Met	2	Alias L., 2004	-	-	-	-
7.1	M	CAV3	c.277G>A	p.Ala93Thr	2	Kubisch C, 2003	c.277G>A	p.Ala93Thr	2	Kubisch C, 2003
8.1	M	CAV3	c.136G>A	p.Ala46Thr	2	Hermann R., 2000	-	-	-	-
8.2	F	CAV3	c.136G>A	p.Ala46Thr	2	Hermann R., 2000	-	-	-	-
8.3	F	CAV3	c.136G>A	p.Ala46Thr	2	Hermann R., 2000	-	-	-	-
9	M	CAPN3	c.1291C>A	p.Val431Met	10	Fanin M., 2004	c.1342C>T	p.Arg448Cys	10	Richard I., 1995
10	F	CAPN3	c.1468C>T	p.Arg490Trp	11	Richard I., 1995	c.1524G>A	Spl?	11	Piluso G., 2005
11	M	CAPN3	c.1469G>A	p.Arg490Gln	11	Dincer P., 1997	c.1469G>A	p.Arg490Gln	11	Dincer P., 1997
12	M	CAPN3	c.1381C>T	p.Arg461Cys	11	Minami N, 1999	c.2242C>T	p.Arg748X	21	Fanin M., 2004
13	M	CAPN3	c.1466G>A	p.Arg489Glu	11	Richard I., 1999	c.1711delC	p.Leu571SerfsX23	13	Guglieri M., 2007
14	M	CAPN3	c.848T>C	p.Met283Thr	6	Fanin M., 2004	c.2115+4delT	Spl?	IVS19	**New**
15	F	CAPN3	c.550delA	p.Thr184fsX36	4	Restagno G., 1996	c.1469G>A	p.Arg490Gln	11	Dincer P., 1997
16	F	CAPN3	c.620A>C	p.Lys207Thr	4	Groen EJ, 2007	c.1746-20C>G	Spl?	IVS13	Piluso G., 2005
17	F	CAPN3	c.535G>C	p.Asp179His	4	Guglieri M., 2007	c.1746-20C>G	Spl?	IVS13	Piluso G., 2005
18	M	CAPN3	c.848T>C	p.Met283Thr	6	Fanin M., 2004	c.2115+4delT	Spl?	IVS19	**New**
19	F	CAPN3	c.1699G>T	Gly567Trp	13	Richard I., 1997	c.1621C>T	p.Arg541Trp	13	Piluso G., 2005
20	F	CAPN3	c.1345A>C	p.Asn449His	10	Fanin M., 2004	c.1345A>C	p.Asn449His	10	Fanin M., 2004
21	F	CAPN3	c.598_612del	p.Phe200-Leu204del	4	Guglieri M., 2007	c.2380+1G>T	exon 22 skippping + activation of a criptic splice site	IVS22	Guglieri M., 2007
22	M	CAPN3	c.801+1G>A	Spl?	IVS5	Haffner K., 1998	c.550delA	p.Thr184ArgfsX36	4	Restagno G., 1996
23	M	CAPN3	c.1468C>T	p.Arg490Trp	11	Richard I., 1995	c.2243G>A	p.Arg748Gln	21	Urtason M., 1998
24	M	CAPN3	c.883_884GA>CT; 887delA	p.Asp295Leu; Asn296Thrfs	6	Guglieri M., 2007	c.2442G>A	p.Trp814X	24	Guglieri M., 2007
25.1	F	CAPN3	c.240C>T	p.Phe80Leu	1	**New**	c.2184G>A	Spl?	20	Piluso G., 2005
25.2	F	CAPN3	c.240C>T	p.Phe80Leu	1	**New**	c.2184G>A	Spl?	20	Piluso G., 2005
26	F	CAPN3	c.1309C>T	p.Arg437Cys	10	Guglieri M., 2007	c.1993-1G>A	Spl?	IVS17	Richard I., 1997
27	M	CAPN3	c.610C>G	p.Leu204Val	4	Nascimbeni AC, 2010	c.1585G>A	p.Ala529Ser	**New**	
28	M	CAPN3	c.1469G>A	p.Arg490Gln	11	Dincer P., 1997	c.1469G>A	p.Arg490Gln	11	Dincer P., 1997
29	F	CAPN3	c.1699G>T	p.Gly567Trp	13	Richard I., 1997	c.1699G>T	p.Gly567Trp	13	Richard I., 1997
30	F	CAPN3	c.2330T>C	p.Ile779Thr	22	Fanin M., 2004	c.550delA	p.Thr184ArgfsX36	4	Restagno G., 1996
31	F	CAPN3	c.1333G>A	p.Gly445Arg	10	Richard I., 1999	c.2380+12delA	Spl?	IVS22	Richard I., 1995

32	M	CAPN3	c.1063C>T	p.Arg355Trp	8	Horvath R., 2005	c.457C>G	p.Gln153Glu	3	**New**
33	F	CAPN3	c.1823G>A	p.Arg608Lys	16	Guglieri M., 2007	c.1992+1G>T	Spl?	IVS17	Leiden database
34	M	CAPN3	c.1468C>T	p.Arg490Trp	11	Richard I., 1995	**c.1194-9A>G**	Spl?	IVS9	Krahn M, 2006
35		CAPN3	c.550delA	p.Thr184fsX36	4	Restagno G., 1996	c.2439+2G>T	Spl?	IVS23	**New**
36.1	F	CAPN3	c.328C>T	p.Arg110X	2	Richard I., 1999	-	-	-	-
36.2	F	CAPN3	c.328C>T	p.Arg110X	2	Richard I., 1999	-	-	-	-
37	M	CAPN3	c.2248C>T	p.Arg748X	21		-	-	-	-
38	F	DYSF	c.757C>T	p.Arg253Trp	7	Nguyen K., 2005	c.90delA	p.Lys33ArgfsX9	2	**New**
39	F	DYSF	c.5529G>A	p.Trp1843X	50	Guglieri M., 2007	c.5529G>A	p.Trp1843X	50	Guglieri M., 2007
40	M	DYSF	c.1911C>A	p.Tyr637X	20	Guglieri M., 2007	c.1911C>A	p.Tyr637X	20	Guglieri M., 2007
41	M	DYSF	c.5594delG	p.Gly1865Ala fsX100	50	Nguyen K., 2005	c.5594delG	p.Gly1865Ala fsX100	50	Nguyen K., 2005
42	M	DYSF	c.2875C>T	p.Arg959Trp	27	Cagliani R., 2005	c.2875C>T	p.Arg959Trp	27	Cagliani R., 2005
43	F	DYSF	c.457+2T>G	Skipping exon 5	IVS5	Cagliani R., 2005	c.2875C>T	p.Arg959Trp	27	Cagliani R., 2005
44.1	F	DYSF	c.2875C>T	p.Arg959Trp	27	Cagliani R., 2005	c.4003G>A	p.Glu1335Lys	37	Cagliani R., 2003
44.2	F	DYSF	c.2875C>T	p.Arg959Trp	27	Cagliani R., 2005	c.4003G>A	p.Glu1335Lys	37	Cagliani R., 2003
45	F	DYSF	c.5626G>A	p.Asp1876Asn	51	Leiden database	c.5626G>A	p.Asp1876Asn	51	Leiden database
46	F	DYSF	c.1120G>C	p.Val374Leu	12	Cagliani R., 2005	c.5033G>C	p.Cys1678Ser	45	Cagliani R., 2005
47	F	DYSF	c.2200-2204del	p.Trp734ProfsX17	23	Aoki M., 2001	c.5303G>T	p.Arg1768Leu	47	**New**
48	M	DYSF	c.2875C>T	p.Arg959Trp	27	Cagliani R., 2005	c.2875C>T	p.Arg959Trp	27	Cagliani R., 2005
49	M	DYSF	c.1254delC	p.Pro418ProfsX123	13	Guglieri M., 2007	c.1254delC	p.Pro418ProfsX123	13	Guglieri M., 2007
50	M	DYSF	c.1254delC	p.Pro418ProfsX123	13	Guglieri M., 2007	c.1254delC	p.Pro418ProfsX123	13	Guglieri M., 2007
51	F	DYSF	c.1639delG	fs con stop ex 20	19	**New**	c.3191-3196dup	fs	30	Therrien C, 2006
52.1	M	DYSF	c.2200_2204del	p.Trp734ProfsX17	23	Aoki M., 2001	c.2200_2204del	p.Trp734ProfsX17	23	Aoki M., 2001
52.2	F	DYSF	c.2200_2204del	p.Trp734ProfsX17	23	Aoki M., 2001	c.2200_2204del	p.Trp734ProfsX17	23	Aoki M., 2001
53.1	F	DYSF	c.2494C>T	p.Gln832X	24	Guglieri M., 2007	c.2494C>T	p.Gln832X	24	Guglieri M., 2007
53.2	M	DYSF	c.2494C>T	p.Gln832X	24	Guglieri M., 2007	c.2494C>T	p.Gln832X	24	Guglieri M., 2007
54.1	M	DYSF	c.2875C>T	p.Arg959Trp	27	Cagliani R., 2005	c.5813_5821del	p.Thr1938Lys del1939-1941 Ala-Lys-Lys	52	Nguyen K., 2005
54.2	M	DYSF	c.2875C>T	p.Arg959Trp	27	Cagliani R., 2005	c.5813_5821del	p.Thr1938Lys del1939-1941 Ala-Lys-Lys	52	Nguyen K., 2005
55.1	M	DYSF	c.3113G>A	p.Arg1038Gln	29	Cagliani R., 2005	c.5979InsA	p.Glu1994ArgfsX3	53	Anderson DY, 2000
55.2	M	DYSF	c.3113G>A	p.Arg1038Gln	29	Cagliani R., 2005	c.5979InsA	p.Glu1994ArgfsX3	53	Anderson DY, 2000
56.1	F	DYSF	c.331C>T	p.Gln111X	4	Leiden database	c.331C>T	p.Gln111X	4	Leiden database
56.2	F	DYSF	c.331C>T	p.Gln111X	4	Leiden database	c.331C>T	p.Gln111X	4	Leiden database
57.1	F	DYSF	c.94A>T	p.Lys32X	2	**New**	c.5440C>T	p.Arg1814Cys	49	**New**
57.2	F	DYSF	c.94A>T	p.Lys32X	2	**New**	c.5440C>T	p.Arg1814Cys	49	**New**
58	F	DYSF	c.2875C>T	p.Arg959Trp	27	Cagliani R., 2005	c.2875C>T	p.Arg959Trp	27	Cagliani R., 2005
59	F	DYSF	c.2000-2004del	p.Thr734ProfsX17	23	**New**	c. 2000-2004del	p.Thr734ProfsX17	23	**New**
60	M	DYSF	c.6233-6240delCCTTCAGC	p.Pro2078Leufs	55*	**New**	c.6233-6240delCCTTCAGC	p.Pro2078Leufs	55*	**New**
61	F	DYSF	c.3950A>G	p.Arg1317Ser	37	**New**	c.3950A>G	p.Arg1317Ser	37	**New**
62	F	DYSF	c.1254delC	p.Pro418ProfsX123	13	Guglieri M., 2007	c.1020C>A	p.Ser340Arg	11	Nguyen K., 2005
63.1	F	DYSF	c.1252G>A	p.Gly618Arg	20	**New**	-	-	-	-
63.2	M	DYSF	c.1252G>A	p.Gly618Arg	20	**New**	-	-	-	-
64	F	DYSF	c.1064_1065del	p.Lys355ArgfsX4	12	Krahn M.2009	-	-	-	-
65	M	SGCG	c.551T>G	p.Val184Gly	6	Guglieri M., 2007	c.551T>G	p.Val184Gly	6	Guglieri M., 2007
66	M	SGCG	c.525delT	p.Phe175LeufsX20	6	Ben Othmane K, 1992	c.525delT	p.Phe175LeufsX20	6	Ben Othmane K, 1992
67	F	SGCG	c.307C>G	p.Leu103Val	3	Guglieri M., 2007	c.551T>G	p.Val184Gly	6	Guglieri M., 2007
68	M	SGCG	c.848G>A	p.Cys283Tyr	8	Piccolo F, 1996	c.848 G>A	p.Cys283Tyr	8	Piccolo F, 1996
69	M	SGCG	c.525delT	p.Phe175LeufsX20	6	Ben Othmane K, 1992	c.525delT	p.Phe175LeufsX20	6	Ben Othmane K, 1992

70	M	SGCA	c.229C>T	p.Arg77Cys	3	Duggan DJ, 1997	c.241C>T	p.Arg81Ser	3	Guglieri M., 2007
71.1	F	SGCA	c.850C>T	p.Arg284Cys	7	Duggan DJ, 1997	c.850C>T	p.Arg284Cys	7	Duggan DJ, 1997
72.1	M	SGCA	c.850C>T	p.Arg284Cys	7	Duggan DJ, 1997	c.850C>T	p.Arg284Cys	7	Duggan DJ, 1997
72.2	F	SGCA	c.850C>T	p.Arg284Cys	7	Duggan DJ, 1997	c.850C>T	p.Arg284Cys	7	Duggan DJ, 1997
73.1	M	SGCA	c.241C>T	p.Arg81Cys	4	Guglieri M., 2007	c.850C>T	p.Arg284Cys	7	Duggan DJ, 1997
73.2	M	SGCA	c. 241 C>T	p.Arg81Cys	4	Guglieri M., 2007	c.850C>T	p.Arg284Cys	7	Duggan DJ, 1997
74	F	SGCA	c.739G>A	p.Val247Met	6	Moreira ES, 2003	c.739G>A	p.Val247Met	6	Moreira ES, 2003
75.1	F	SGCA	c.271G>A	p.Gly91Ser	3	Guglieri M., 2007	c.292C>T	p.Arg98Cys	3	Ljunggren A., 1995
75.2	F	SGCA	c.271G>A	p.Gly91Ser	3	Guglieri M., 2007	c.292C>T	p.Arg98Cys	3	Ljunggren A., 1995
76	M	SGCA	c.307A>T	p.Ile307Trp	3	Guglieri M., 2007	c.307A>T	p.Ile307Phe	3	Guglieri M., 2007
77	M	SGCA	c.622A>G	p.Thr208Ala	3	Guglieri M., 2007	c.622A>G	p.Thr208Ala	3	Guglieri M., 2007
78	M	SGCA	c.850C>T	p.Arg284Cys	7	Duggan DJ, 1997	c.850C>T	p.Arg284Cys	7	Duggan DJ, 1997
79	F	SGCA	c.850C>T	p.Arg284Cys	7	Duggan DJ, 1997	c.850C>T	p.Arg284Cys	7	Duggan DJ, 1997
80	M	SGCA	c.935T>G	p.Met312Arg	7	Guglieri M., 2007	c.935T>G	p.Met312Arg	7	Guglieri M., 2007
81.1	F	SGCA	c.739G>A	p.Val247Met	6	Moreira ES, 2003	c.739G>A	p.Val247Met	6	Moreira ES, 2003
81.2	F	SGCA	c.739G>A	p.Val247Met	6	Moreira ES, 2003	c.739G>A	p.Val247Met	6	Moreira ES, 2003
81.3	F	SGCA	c.739G>A	p.Val247Met	6	Moreira ES, 2003	c.739G>A	p.Val247Met	6	Moreira ES, 2003
82	F	SGCB	c.341C>T	p.Ser114Phe	3	Duggan DJ, 1997	c.341C>T	p.Ser114Phe	3	Duggan DJ, 1997
83.1	F	SGCB	c.377_384dup	p.Gly128GlnfsX2	3	Duggan DJ, 1997	c.377_384 dup	p.Gly128GlnfsX2	3	Duggan DJ, 1997
83.2	M	SGCB	c.377_384dup	p.Gly128GlnfsX2	3	Duggan DJ, 1997	c.377_384dup	p.Gly128GlnfsX2	3	Duggan DJ, 1997
84	F	SGCB	c.377_384dup	p.Gly128GlnfsX2	3	Duggan DJ, 1997	c.377_384 dup	p.Gly128GlnfsX2	3	Duggan DJ, 1997
85	M	SGCB	c.85A>T	p.Arg29X	2	Cagliani R, 2001	c.271C>T	p.Arg91Cys	3	Duclos F, 1998
86	F	SGCB	c.377_384dup	p.Gly128GlnfsX2	3	Duggan DJ, 1997	c.377_384dup	p.Gly128GlnfsX2	3	Duggan DJ, 1997
87	M	SGCD	c.382+1_382+2del	Spl?	IV5	Guglieri M., 2007	c.382+1_382+2del	spl?	IV5	Guglieri M., 2007
88	F	FKRP	c.826C>A	p.Leu276Ile	4	Brockington M, 2001	c.1325C>T	p.Pro442Leu	4	Guglieri M., 2007
89.1	M	FKRP	c.826C>A	p.Leu276Ile	4	Brockington M, 2001	c.1087G>C	p.Val363Leu	4	Leiden database
89.2	M	FKRP	c.826C>A	p.Leu276Ile	4	Brockington M, 2001	c.1087G>C	p.Val363Leu	4	Leiden database
90	M	FKRP	c.826C>A	p.Leu276Ile	4	Brockington M, 2001	c.826C>A	p.Leu276Ile	4	Brockington M, 2001
91	M	FKRP	c.826C>A	p.Leu276Ile	4	Brockington M, 2001	c.826C>A	p.Leu276Ile	4	Brockington M, 2001
92	F	FKRP	c.826C>A	p.Leu172Ile	4	Brockington M, 2001	c.826C>A	p.Leu172Ile	4	Brockington M, 2001
93	M	FKRP	c.826C>A	p.Leu276Ile	4	Brockington M, 2001	c.826C>A	p.Leu276Ile	4	Brockington M, 2001
94	M	FKRP	c.956T>G	p.Leu319Arg	4	Guglieri M., 2007	c.1136delG	p.Arg379ArgfsX10	4	Guglieri M., 2007
95	M	FKRP	c.826C>A	p.Leu276Ile	4	Brockington M, 2001	c.993insC	p.Val332ArgfsX57	4	**New**
96	M	ANO5	c.397A>T	p.Ile133Phe	7	Magri F, 2012	c.397A>T	p.Ile133Phe	7	Magri F, 2012
97	M	ANO5	c.220C>T	p.Arg74X	5	Magri F, 2012	c.1609T>C	p.Ser537Pro	15	Magri F, 2012
98.1	M	ANO5	c.1627dupA	p.Met543AsnfsX10	15	Magri F, 2012	c.1627dupA	p.Met543AsnfsX10	15	Magri F, 2012
98.2	M	ANO5	c.1627dupA	p.Met543AsnfsX10	15	Magri F, 2012	c.1627dupA	p.Met543AsnfsX10	15	Magri F, 2012
99.1	M	ANO5	c.191dupA	p.Asn64LysfsX15	5	Brais B, 2010	c.2516T>G	p.Met839Arg	21	Magri F, 2012
99.2	F	ANO5	c.191dupA	p.Asn64LysfsX15	5	Brais B, 2010	c.2516T>G	p.Met839Arg	21	Magri F, 2012
100	M	ANO5	c.817C>T	p.Leu273Phe	9	Linssen WH, 2013	c.2387C>T	p.Ser796Leu	20	Sarkozy A, 2012
101	M	LAMA2	c.6742delC	p.Leu2248TrpfsX23	48	**New**	c.8544C>G	p.His2848Gln	60	**New**
102	M	ISPD	c.676T>C	p.Tyr226His	3	Vuillaumier-Barrot, 2012	c.836-5T>G	Spl?	IVS5	**New**
